# Supplementary material for: Asynchronous magnetic resonance elastography: Shear wave speed reconstruction using noise correlation of incoherent waves
Source: Magn Reson Med. 2022 Oct 27;89(3):990–1001. doi: 10.1002/mrm.29502 (PMC9792433; doi:10.1002/mrm.29502)
Supplement: Supplementary file 1 — DATA S1 MATLAB code used to generate the numerical simulations of Figure 1 [file MRM-89-990-s002.zip › k-Wave/helpfiles/waterDensity.html]

waterDensity :: Functions (k-Wave)


# waterDensity

Calculate density of air-saturated water with temperature.

## Syntax

```
density = waterDensity(T)
```

## Description

`waterDensity` calculates the density of air-saturated water at a given temperature using the 4th order polynomial given by Jones (1992, ITS-90 Density of Water Formulation for Volumetric Standards Calibration, J. Res. Natl. Inst. Stand. Technol., 97(3), 335-340).

## Inputs

|  |  |
| --- | --- |
| `T` | water temperature in the range 5 to 40 [degC] |

## Outputs

|  |  |
| --- | --- |
| `density` | density of water [kg/m^3] |

## See Also

`waterAbsorption`, `waterNonlinearity`, `waterSoundSpeed`
